# Unveiling the Development of Sprint Athletes: Percentile Patterns, Peak Performance Age, and a Performance Progression Model

**DOI:** 10.5114/jhk/187621

**Published:** 2024-07-17

**Authors:** Aarón Agudo-Ortega, Jesús Santos del Cerro, Juan J. Salinero, José M. González-Rave

**Affiliations:** 1 Sports Training Laboratory, Faculty of Sports Sciences, University of Castilla-La Mancha, Toledo, Spain.; 2 Department of Economics and Business Statistics, University of Castilla-La Mancha, Toledo, Spain.

**Keywords:** track-and-field, sprint, percentile curves, peak age, performance trajectories

## Abstract

The purpose of this study was to provide percentile curves, peak performance age for all sprint distances, and two linear regression models in order to analyse the individual trajectories of Spanish sprinters to explain the achievement of the senior category. A retrospective analysis was undertaken using rankings of the Spanish Athletics Federation. We analysed 4398 sprint athletes between 2004 and 2021. Our results show that the percentile curves are farther apart as the distance increased. Peak performance age was reached earlier in males than females in all distance categories (25.31 ± 0.12 and 25.79 ± 0.70 years for 100 m, 25.45 ± 0.16 and 27.40 ± 0.31 years for 200 m, and 25.61 ± 0.24 and 27.46 ± 2.28 years for 400 m in males and females, respectively). The two linear regressions display the importance of consistent high performance in junior categories (p < 0.01; β = −1.92 and p = 0.15; β = −1.22, respectively) together with the best results in the U23 category (p < 0.001; β = 0.51 and p < 0.001; β = 0.51, respectively) to achieve participation in the senior category. We conclude that as the running distance increases, the differences between percentiles also increase, the peak performance age occurs earlier in Spanish sprinters than shown in previous research for all sprint distances, and attaining the senior category depends upon achieving the best results in previous categories and gaining extensive experience (consistent high-performance participation) in the event.

## Introduction

The development and progression of athletes, as well as their future potential, encompass multifactorial aspects (De Larochelambert et al., 2023) aimed at achieving elite performance. Consequently, developing organic systems (Gorzi et al., 2022) coupled with accurate training periodisation (González-Ravé et al., 2022; Stone et al., 2021) and planning (Agudo-Ortega et al., 2024; Krolikowska et al., 2023; Loturco et al., 2023a, 2023b) becomes crucial for maximising athletic performance. During early stages, the processes of growth, maturation, and development occur simultaneously, exerting an influence on physical activity, fitness, and performance (Malina et al., 2010; Malina, 2014). The correct development of these three processes could help athletes to acquire the necessary motor competence within a specific timeframe (Živković et al., 2022) and should be considered within the talent identification and selection system. The process of talent detection in sports remains significantly biased towards competition outcomes, particularly in sports measured in centimetres, grams or seconds, such as athletics (Moesch et al., 2011). The current literature highlights the substantial error in talent identification based solely on early-age results due to the relative age effect (Brazo-Sayavera et al., 2017; Hollings et al., 2014a; Kalén et al., 2021) and low transition rates between junior and senior categories (Agudo-Ortega et al., 2023; Boccia et al., 2019; Güllich et al., 2023; Kearney and Hayes, 2018).

Experts need to determine sport-specific requirements (power, technique and sprint-specific endurance) for sprint athletes (Haugen et al., 2019) to achieve the correct development and training periodization and programming at each stage of a career in track-and-field events. These specific demands in these events are based on the “window” for peak performance and peak performance ages (Haugen et al., 2018; Hollings et al., 2014b). Developmental differences between sexes have been recorded in favour of boys from 6–7 years, the disparity increasing in strength and power sports at the age of 12 (Dobosz et al., 2015; Steenman et al., 2016). Despite a trend toward better performance, girls reach a plateau between 13 and 15 years, and boys between 16 and 18 years (Dobosz et al., 2015; Tønnessen et al., 2015).

Regarding peak performance age, Elmenshawy et al. (2015) have shown that this age in 100 m running has progressively increased over the successive Olympic Games. In the international track-and-field domain, Boccia et al. (2021) have analysed the peak performance age in jumping events in men and women finding the ages to be 25.8 and 26.3 years in the high jump as well as the triple jump, 25.0 and 26.8 years in the long jump, and 26.0 and 27.1 years in the pole vault, respectively. Similarly, peak performance ages were also analysed in international sprint events for men and women, the ages being 22.6 and 22.3 years in the 100 m, 23.1 and 22.6 years in the 200 m, and 21.2 and 24.7 years in the 400 m event, respectively (Boccia et al., 2020). At the national level, the age of peak performance has been analysed in Italian track-and-field events, showing ages for males and females of 21.6 and 21.5 years in the high jump, 23.4 and 21.5 years in the long jump (Boccia et al., 2017), 24.7 and 24.7 years in the discus throw, 23.5 and 24.5 years in the shot put, 25.3 and 24.5 years in the hurdles, and 23.4 and 23.6 years in sprint events, respectively (Boccia et al., 2019).

Performance trajectories for individuals have received limited attention from the scientific community. Haugen et al. (2018) have analysed the performance progression of world-class track-and- field athletes, revealing more substantial improvements in the five years leading up to their peak performance age in athletes performing the 100 m sprint. Tønnessen et al. (2015), examining performance progression in track-and-field athletes during development years, have highlighted increases of 0.3–0.5 s under 14 years, from 0.1 to 0.2 s between 14 and 17 years and 0.1–0.2 s from 17 to 18 years in sprinters. Boccia et al. (2017), studying the annual rate of change from Italian long and high jumpers, have reported greater change rates at the top level compared to the rest of athletes before reaching peak performance age. Weippert et al. (2021) have analysed performance trajectories for athletes in middle-distance runners with the World Record time, revealing a higher relative performance level among females at the beginning, and a higher relative performance level at later stages among males.

Current research indicates that early-stage success is not a prerequisite for success in the senior category of track-and-field events, that is, high performance in junior categories does not define an athlete’s future performance at senior levels of competition (Agudo-Ortega et al., 2023; Barth et al., 2022; Kliethermes et al., 2020). Furthermore, research has also emphasized the utility of percentile curves in comprehensively covering performance progression across the entire spectrum of performance levels. These percentile curves, in conjunction with normative data, offer reference values for each year or category, facilitating the identification of performance progression above or below the average between years, and identify individual strengths and weaknesses (Born et al., 2022).

In summary, the existing research has elucidated different peak performance ages and trajectories for individuals across different stages in sports (Boccia et al., 2019, 2020; Haugen et al., 2018; Tønnessen et al., 2015) in addition to establishing normative data and percentile values in individual sports (i.e., swimming) (Born et al., 2022). However, no studies available to date have provided these percentile curves in track-and-field athletes and no evidence is yet available for peak performance ages and individual trajectories at the national level in sprinters. Therefore, this study provides percentile curves (from the junior to the senior category) and peak performance ages for the different sprint distances (100 m, 200 m, 400 m), as well as two linear regressions in order to analyse the individual trajectories of Spanish sprinters that explain the achievement of the senior category.

## Methods

### Participants

Analyses were made for a total of 4398 sprinters with 28146 records who appeared in the Spanish Athletics Federation ranking database during the period from 2004 to 2021.

### Design and Procedures

A retrospective analysis was undertaken to examine the percentile curves, peak performance age, and individual trajectories of Spanish sprinters.

The data were acquired from the public database of the Spanish Athletics Federation (RFEA), https://www.rfea.es/web/estadisticas/ranking.asp (access date: 07 February 2022), compiling the best personal times of the different athletic competitions from the U14 to the senior category in Spain since the 2004/2005 season. The data were divided by sex, the type (outdoor events), the season, the category, and a specific event, including both manual and electronic times and both legal and illegal wind. For all athletes who had competed in Spain each season, the athlete's name was recorded, together with the ranking, the mark, the date of birth, and the category, among other data. Due to the open data source employed for the data collection, approval by the local ethics committee was not required.

All data from the 2004/2005 to 2021 seasons for all categories (from U14 in 100 m, and U16 in 200 m and 400 m) was downloaded in Microsoft Excel spreadsheets for 100 m, 200 m, and 400 m athletes. To ensure data integrity, we excluded from the database any results obtained with illegal wind (+2 m/s), and athletes of other nationalities.

The times were standardized employing Ztime scores to compare athletes’ times without influencing the variables of sex, the category or distance. The Ztime score was created using the performance of each athlete.


$$Z_{ij}=\frac{x_{ij}-{\bar{x}}_{ij}}{\sigma_{i}}$$

where j = individual, while i = group by sex, category, and distance. This standardized approach enhances the accuracy of comparisons and lends itself to more robust analyses of performance trends and patterns across diverse athletic disciplines.

We selected each athlete with the best mark (in terms of Ztime) in each established junior (U14, U16, U18, U20, U23) and senior category. We considered athletes who participated at least five times in three or more junior categories. For athletes who did not participate in any junior category, we applied interpolation techniques based on linear regressions over their best mark (e.g., for one athlete who participated in U14, U18, and U23, we estimated the linear regression, and the estimates were interpolated for U16 and U20).

### Statistical Analysis

The percentile curve and peak performance age are presented as mean ± SD per distance and sex.

The Decision Tree classification method with the machine learning technique was used to explain which results in the junior categories proved the most influential in the achievement of and participation in the senior category.

Linear regression was used to explain the best marks that depended on the previous trajectories and the consistent high-performance participation (reaching the top 50 status) (P_e_). This model indicates the importance of some predictors (independent variables such as a category or a personal best (PB)) on the dependent variable (Ztime) when considering the best athletes that appeared in the junior and senior Spanish ranking. We generated two linear regression models to explain the best scores in the senior category as a function of the normalized times in each of the categories reported by the Ztime. The first model was based on athletes who competed in at least three categories, while the second was for athletes who competed in four categories or more.

Statistical analyses were conducted using R software (v. 4.3.0 for Windows).

## Results

**Figure 1:**
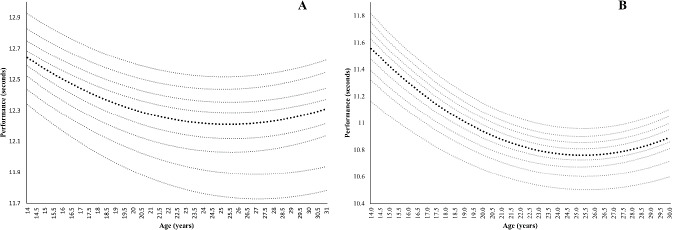
Percentile curves and peak performance age for 100 m for female (A) and male (B) Spanish sprinters.

Figure 1 shows percentile curves from 100 m for females and males. The age of peak performance for males was 25.31 ± 0.12 years and for females 25.79 ± 0.70. No significant differences were found (*p* = 0.07) between sexes.

**Figure 2:**
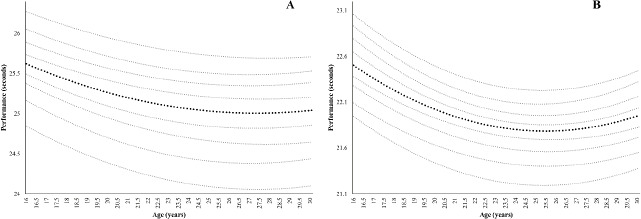
Percentile curves and peak performance age for 200 m for female (A) and male (B) Spanish sprinters.

In addition, Figure 2 shows percentile curves from 200 m for females and males. The age of peak performance for males was 25.45 ± 0.16 years and for females 27.40 ± 0.31 years with a significant difference between sexes (*p* < 0.001).

**Figure 3:**
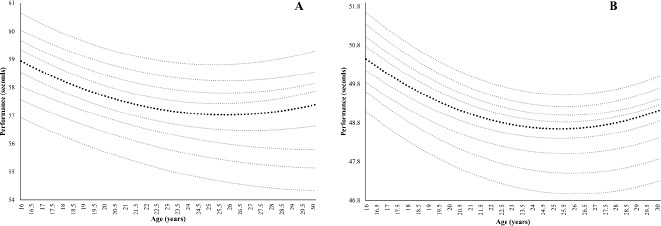
Percentile curves and peak performance age for 400 m for female (A) and male (B) Spanish sprinters.

Furthermore, Figure 3 shows percentile curves from 400 m for females and males. The age of peak performance in males was 25.61 ± 0.24 years and for females 27.46 ± 2.28. As in the 200 m event, the difference between sexes was significant (*p* = 0.004).

The coefficients for the linear regression model, based on athletes who competed in three categories, are presented in Table 1. This linear regression showed statistical significance in the F-test (*p* < 0.001). The adjustment of the coefficient of R^2^ was 0.43, suggesting an adequate “degree of goodness” and consequently a model with strong explanatory power. In this regression model, the most influential variable was U23 PB (*p* < 0.001; β = 0.51) with a β value signifying that a one-point improvement in the U23 PB variable corresponds to a 0.51 improvement in the explained variable (Sen PB). Furthermore, significant differences appeared in U18 PB (*p* = 0.03; β = −0.20), U20 PB (*p* = 0.03; β = 0.15) and P_e_ (*p* < 0.001; β = −1.92).

**Table 1:** Coefficients for the linear regression (LR) model of participants who competed in three categories. * *p* < 0.01; ** *p* < 0.001; *** *p* < 0.0001

Coefficients	Estimate (β)	Std. Error	Z value	*p* value
(Intercept)	25.86	14.43	1.79	0.07*
U14_PB	0.10	0.08	1.26	0.20
U16_PB	−0.11	0.10	−1.09	0.27
U18_PB	−0.20	0.09	−2.10	0.03*
U20_PB	0.15	0.07	2.09	0.03*
U23_PB	0.51	0.08	6.18	2.87e-09***
P_e_	−1.92	0.57	−3.33	0.00**

The coefficients for the linear regression model based on athletes who competed in four categories or more are presented in Table 2. This linear regression shows statistical significance in the F-test (*p* < 0.001). The adjustment of the coefficient R^2^ was 0.43, suggesting an adequate “degree of goodness” and consequently a model with adequate explanatory power. As in the previous regression model, U23 PB (*p* < 0.001; β = 0.51) was the most influential variable in this regression. This β value means that each one-point improvement in the variable U23 PB implies a 0.51 improvement in the explained variable (Sen PB). Also, significant differences were found in U20 PB (*p* = 0.09; β = 0.18) and P_e_ (*p* = 0.15; β = −1.22).

**Table 2:** Coefficients for the linear regression (LR) model of participants who competed in four categories or more. *p < 0.01; **p < 0.001; ***p < 0.0001

Coefficients	Estimate (β)	Std. Error	Z value	*p* value
(Intercept)	26.34	25.85	1.01	0.31
U14_PB	0.18	0.09	2.00	0.04*
U16_PB	−0.13	0.10	−1.20	0.23
U18_PB	−0.17	0.10	−1.65	0.10
U20_PB	0.18	0.10	1.70	0.09
U23_PB	0.51	0.11	4.63	1.28e-05***
P_e_	−1.22	0.86	−1.42	0.15

**Figure 4:**
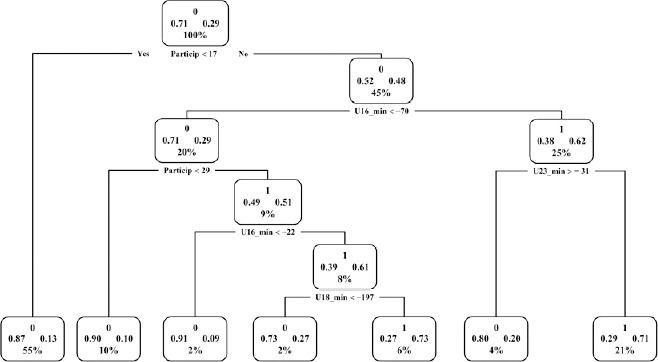
Decision-tree method. Dichotomous variable: athletes ranked in the senior category (1) and athletes not ranked in the senior category (0). The following variables were analysed for statistical significance: U14_PB: best mark in the U14 category; U16_PB: best mark in the U16 category; U18_PB: best mark in the U18 category; U20_PB: best mark in the U20 category; U23_PB: best mark in the U23 category; Pe: consistent high-performance participation.

Deviation analyses using the decision-tree method revealed that achieving the senior category could be attributed to two types of circumstances: a) consistent high performance in junior categories (reaching top 50 status more than 17 times in junior categories) and achieving good results in the U23 category; or b) consistent high performance in junior categories (reaching top 50 status more than 30 times in junior categories) and obtaining good results in the U16 and U18 categories, as illustrated in Figure 4. The results of the prediction model have an accuracy of 74%.

## Discussion

The purpose of this study was to provide percentile curves, peak performance age and two linear regressions in order to analyse the individual trajectories of Spanish sprinters. The data showed that the percentiles were farther apart as the distance increased, and the age for peak performance occurred significantly earlier in males than in females in 200 m and 400 m, while the linear regressions indicated that consistent high-performance in junior categories and the best results in some junior categories could predict good results in the senior category.

The percentile curves in Figures 1–3 demonstrate that as the running distance increased, the differences between percentiles also increased, and these differences were more noticeable in females than males. These results are in line with the work of Born et al. (2022) with percentile curves in swimmers. That is, males achieved better marks in all activities. This may be attributed to the fact that during adolescence, boys experienced more rapid acceleration in their improvements (the growth spurt in strength doubling that of girls), while girls improved only slightly or levelled off (Malina, 2014). At the same time, when the loss of performance phase began, this loss was less pronounced in females than males at all distances. Conversely, our results differ from previous research on master athletes that shows greater declines in performance rates in females than in males for sprint events (Ganse et al., 2020; Wright and Perricelli, 2008).

In the international field, Haugen et al. (2018) have found similar peak performance ages among world-class sprinters to our results in males and females. On the other hand, Boccia et al. (2020) reported peak performance ages of four different groups divided by sex: only U18 (ranked top 50 in U18 but not in senior), U18 and senior (top ranked in U18 and senior), only senior (ranked top 50 in senior but not in U18) and others (not ranked in top 50 in any category) reporting different peak performance age than in Spanish sprinters. Such discrepancies in results appear because of the different methodological approaches applied in the studies. Our study used the complete Spanish Athletics Federation ranking database with standardized categories (U14, U16, U18, U20, U23 and senior category), while Boccia et al. (2020) created the four categories previously mentioned. Therefore, future studies are needed with a standardized methodological protocol for comparing these results. A similar pattern occurs when we focus on the national field. Boccia et al. (2019) presented data from Italian 100 m sprinters who achieved peak performance at an earlier age than Spanish sprinters (25.31 ± 0.12 vs. 23.4 ± 3.3 in males and 25.79 ± 0.70 vs. 23.6 ± 3.7 in females in Spanish and Italian sprinters, respectively). Just as in the international context, the methodological factor influences this peak performance age.

Concerning performance progression models, this is the first study to conduct this type of retrospective analysis with a linear regression model of sprint athletes. Our results suggest an adequate “degree of goodness”, even though the adjustment of the coefficient R^2^ was 0.43 because it must be taken into account that the model did not include variables related to training or physiological characteristics, as well as nutrition or rest that would provide a better goodness-of-fit of the model. Previous authors have used other types of performance progression models in track-and-field events. Weippert et al. (2021) used a performance progression model to compare individual trajectories of 800 m and 1500 m athletes with the world-record time. In contrast, Boccia et al. (2017) and Haugen et al. (2018) used this linear regression to compare success between the elite (percentile between 97 and 100 and the top 10, respectively) and their counterparts at lower performance levels. In our case, we used this regression to identify the circumstances needed to achieve the senior category.

The results of our two lineal regressions highlight the significance of experience (consistent high-performance participation in junior categories) combined with the best results in the U23 category (among other junior categories) to achieve participation in the senior category. This aligns with the findings of Rees et al. (2016) who showed that super-elite athletes accumulated a high amount of organized practice and training, with significant differences in late adolescence, whereas Boccia et al. (2017) indicated that Italian jumpers entered competition early (eight years before the senior category) with frequent participation and extensive experience in this discipline, reinforcing the idea that wealth of experience is an important factor to achieve the senior category.

The last systematic reviews and meta-analyses in this field (Barth et al., 2024; Güllich et al., 2023) illustrate that the two categories prior to the senior stage had a higher correlation for success in this category in individual sports, reinforcing the results of our two linear regressions. These outcomes reinforce the idea of late specialisation, as described by several previous studies (Agudo-Ortega et al., 2023; Barth et al., 2022, 2024; Güllich et al., 2023) and reaffirm the idea of a sports developmental model based on late specialisation, such as the Developmental Model of Sport Participation proposed by Côté (1999). It has been noted that the transition rate (top 20) between U23 and the senior category is a harder overstep in Spanish sprinters (Agudo-Ortega et al., 2023). However, our results demonstrate that high performance in this U23 category is a determinant for a sprinter to reach the senior category. For this reason, qualitative research is needed with elite athletes to determine more accurately what their sporting development towards the elite has been.

## Conclusions

Based on the results, we may conclude according to Spanish track-and-field data, percentile curves are farther apart as the distance increases following the same pattern as in other sports (e.g., swimming). The peak performance age occurs earlier among Spanish sprinters than among their international and other national counterparts in all three distances (100 m, 200 m, and 400 m) with significant differences between sexes in 200 m and 400 m. Additionally, achieving the senior category depends upon reaching the best results in the previous categories and accumulating substantial experience (in terms of consistent high performance) in the event.

It bears mentioning that this study relies on quantitative research utilizing data from the Spanish Athletics Database Rankings. However, it may not provide a comprehensive explanation for the entire trajectory of Spanish athletes, as this would require qualitative research on these athletes’ progression, which, together with this quantitative study, would provide an overall view of the performance progression of elite athletes in sprint performance.
